# Toward a New U.S. Chemicals Policy: Rebuilding the Foundation to Advance New Science, Green Chemistry, and Environmental Health

**DOI:** 10.1289/ehp.0800404

**Published:** 2009-02-09

**Authors:** Michael P. Wilson, Megan R. Schwarzman

**Affiliations:** Center for Occupational and Environmental Health, School of Public Health, University of California at Berkeley, Berkeley, California, USA

**Keywords:** chemicals policy, data gap, environmental health, green chemistry, innovation, REACH, safety gap, sustainability, technology gap, TSCA

## Abstract

**Objective:**

We describe fundamental weaknesses in U.S. chemicals policy, present principles of chemicals policy reform, and articulate interdisciplinary research questions that should be addressed. With global chemical production projected to double over the next 24 years, federal policies that shape the priorities of the U.S. chemical enterprise will be a cornerstone of sustainability. To date, these policies have largely failed to adequately protect public health or the environment or motivate investment in or scientific exploration of cleaner chemical technologies, known collectively as green chemistry. On this trajectory, the United States will face growing health, environmental, and economic problems related to chemical exposures and pollution.

**Conclusions:**

Existing policies have produced a U.S. chemicals market in which the safety of chemicals for human health and the environment is undervalued relative to chemical function, price, and performance. This market barrier to green chemistry is primarily a consequence of weaknesses in the Toxic Substances Control Act. These weaknesses have produced a chemical data gap, because producers are not required to investigate and disclose sufficient information on chemicals’ hazard traits to government, businesses that use chemicals, or the public; a safety gap, because government lacks the legal tools it needs to efficiently identify, prioritize, and take action to mitigate the potential health and environmental effects of hazardous chemicals; and a technology gap, because industry and government have invested only marginally in green chemistry research, development, and education. Policy reforms that close the three gaps—creating transparency and accountability in the market—are crucial for improving public and environmental health and reducing the barriers to green chemistry. The European Union’s REACH (Registration, Evaluation, Authorisation and Restriction of Chemicals) regulation has opened an opportunity for the United States to take this step; doing so will present the nation with new research questions in science, policy, law, and technology.

In the United States, the primary legal framework for managing industrial chemicals used in processes and products, the Toxic Substances Control Act (TSCA 1976), is now over 30 years old and is widely recognized as having failed to meet the intent of Congress. Analyses of TSCA by the National Research Council (NRC 1984), U.S. Government Accountability Office (U.S. GAO 1994, 2005a, 2007a, 2009), Congressional Office of Technology Assessment (OTA 1995), U.S Environmental Protection Agency (U.S. EPA 1998, 2003), Environmental Defense Fund (Denison 2009; Roe et al. 1997), and a former U.S. EPA assistant administrator (Goldman 2002) have concluded that the statute has all but prevented government, businesses, and the public from *a*) assessing the hazard traits of the great majority of chemicals in commerce; *b*) controlling chemicals of significant concern; and *c*) motivating broad industry investment in cleaner chemical technologies and safer alternatives, known collectively as green chemistry.

These outcomes are the consequence of weaknesses in TSCA that have produced three overarching gaps in the U.S. chemicals policy (Figure 1) (Wilson et al. 2006, 2008):

- Data gap: Producers are not required to investigate and disclose sufficient information on the hazard traits of chemicals to government, the public, or businesses that use chemicals.
- Safety gap: Government lacks the legal tools it needs to efficiently identify, prioritize, and take action to mitigate the potential health and environmental effects of hazardous chemicals.
- Technology gap: Industry and government have invested only marginally in green chemistry research, development, and education.

Over the last 30 years, the three gaps have given rise to a U.S. chemicals market that undervalues the safety of chemicals relative to their function, price, and performance. Chemicals and products are bought and sold primarily on the basis of how much work they perform per unit cost, with very little attention given in the market to their potential impacts on human health and ecosystems throughout the chemical lifecycle. Hazardous chemicals have thus remained competitive, and broad industrial investment in green chemistry has lagged, despite efforts of some leading companies. Reflecting these market conditions, the nation’s research and education agendas have neither prioritized green chemistry development nor adequately prepared the next generation of scientists to lead the chemical enterprise toward sustainability. Collectively, the three gaps—and the market conditions they have engendered—present a formidable barrier to the scientific, technical, and commercial success of green chemistry in the United States.

Facing a similar set of chemical management problems—and their consequences for human health and the environment—the European Union in 2006 enacted a sweeping new chemicals regulation known as the Registration, Evaluation, Authorisation and Restriction of Chemicals (REACH) (European Commission 2008; REACH 2007; U.S. Department of Commerce 2008). REACH responds to the barriers described by the data gap and safety gap by requiring producers to disclose some hazard and exposure information on an estimated 30,000 industrial chemicals. Chemical manufacturers must also gain government authorization to use certain “substances of very high concern.” These new requirements—both for data and for proving safe use—are expected to promote the development and use of safer chemical substances, closing the technology gap by fueling new investment in green chemistry science, technology, and education (Black 2008; Buckland 2008; Henzelmann et al. 2007).

Because REACH applies equally in most aspects to manufacturers in the European Union and foreign importers, it is forcing change among chemical and product manufacturers far beyond Europe’s borders (Schapiro 2007). Manufacturers worldwide cannot afford the losses in market share that would result should they fail to comply with REACH.

As U.S. producers prepare hazard and exposure information for the European Union Chemicals Agency (ECHA), the United States has a unique opportunity both to make use of the data and to revisit its own chemicals policy. In doing so, the United States should consider a portfolio of measures that simultaneously close the data, safety, and technology gaps. This approach will most effectively—and with minimal delay—instill within the chemicals market a more appropriate set of incentives and disincentives that are a precondition to motivating broad investment in green chemistry.

As with REACH in the European Union, a new chemicals policy in the United States has the potential to fuel global demand for safer substances and processes, increasing the incentive for research and development in green chemistry while improving human and environmental health. It also could move the United States into a position of greater collaboration in international sustainability efforts and position the country as a global leader in green chemistry innovation. Environmental health scientists have an essential role in identifying and addressing the research questions that will arise with the development of a new U.S. chemicals policy.

## Background

### A key industry

Over the last 150 years, the U.S. chemical industry has contributed significantly to both the national and global economy (Aftalion 2001; Arora et al. 1998). The industry’s contributions to economic growth, employment, and improvements in life expectancy, health, and living conditions in Western-style societies are widely recognized (NRC 1992; Spitz 2003). The industry’s products are ubiquitous; in roughly the last 50 years, synthetic chemicals have become integrated into nearly all industrial processes and commercial products and now constitute the primary material base of society (Geiser 2001).

The scale of chemical production is correspondingly enormous: Data from the TSCA Inventory Update Rule (IUR) show that the United States produced or imported about 15 trillion pounds of chemical substances during the 2002 reporting cycle, or about 42 billion pounds per day (U.S. EPA 2005). For the 2005 reporting period, chemical manufacturers reported producing or importing about 27 trillion pounds of 6,200 chemicals at more than 25,000 pounds per site per year, or about 74 billion pounds per day. The IUR data include substances used in industrial processes and products and do not include fuels, pesticide products, pharmaceuticals, or food products. There is no clear explanation for the 80% increase in volume between 2002 and 2005, and the U.S. EPA has obscured the categories of the products in which each chemical is used (U.S. EPA 2009). The TSCA inventory now lists about 83,000 substances that have been for sale in the United States at some point since the inventory was first published in 1979 (U.S. EPA 2008a). Of these, approximately 62,000 were in commercial use at the time TSCA was passed in 1976, and about 20,000 new substances have entered commercial use since that time (U.S. GAO 2005a).

**Figure 2:**
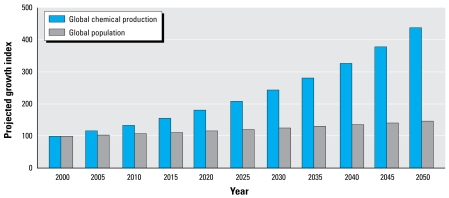
Global chemical production is projected to grow at a rate of 3% per year, rapidly outpacing the rate of global population growth, estimated at 0.77% per year. On this trajectory, chemical production will double by 2024, indexed to 2000 (American Chemistry Council 2003; OECD 2001; United Nations 2004).

Global chemical production is projected to continue growing—about 3% per year, with a doubling rate of 24 years, rapidly outpacing the rate of global population growth (Figure 2) [American Chemistry Council 2003; Organisation for Economic Cooperation and Development (OECD) 2001; United Nations 2004]. This growth will distribute globally both the benefits and the health and environmental consequences of industrial chemical technologies.

### Human and environmental health consequences

#### Bioaccumulative chemicals

Because of their wide distribution throughout the economy and environment, many industrial chemicals come in contact with people: in the workplace, in homes, through the use of products, and via air, water, food, and waste streams. Ultimately, at some point in their life cycle, all industrial chemicals will enter the earth’s ecosystems. Biomonitoring studies are demonstrating widespread human exposure to certain industrial chemicals and pollutants. In 2001–2002, the U.S. Centers for Disease Control and Prevention (CDC) looked for, and found, 148 synthetic chemicals and pollutants in the blood and urine of a representative sample of the U.S. civilian population (CDC 2005). The 2008 assessment is anticipated to include testing for about 250 substances for participants in the 2003–2004 National Health and Nutrition Examination Survey period.

#### Early life exposures

Evidence that many xenobiotic chemicals pass through the placenta, entering and, in some cases, accumulating in the fetus, suggests they could pose significant risks to human development (Barr et al. 2007; Doucet et al. 2008; Grandjean et al. 2007). Rising incidence of some cancers, asthma, and developmental disorders may be due in part to chemical exposures, particularly those that occur during development (Hertz-Picciotto and Delwiche 2009; Sharpe and Irvine 2004; Surveillance, Epidemiology, and End Results Program 2008). A variety of male reproductive abnormalities may also be linked to prenatal exposures to certain pesticides or endocrine-disrupting chemicals (Bay et al. 2006; Main et al. 2006; Skakkebaek 2002; Swan et al. 2000, 2005). The data gap limits investigators’ ability to establish these links, and TSCA has all but prevented the U.S. EPA from instituting more than voluntary measures to act on these early indicators of harm. In assessing the state of the science, the Faroes Statement of the International Conference on Fetal Programming and Developmental Toxicity concluded that efforts to prevent exposures to hazardous chemicals should focus on protecting the embryo, fetus, and small child as highly vulnerable populations. The United States, however, lacks both the information and the regulatory mechanisms necessary to accomplish this goal (Grandjean et al. 2007).

#### Occupational disease

Workers are at particular risk from chemical exposures because, depending on their occupation, they can be more highly exposed to hazardous substances than the general public (LaDou 1997). Although the burden of all-cause occupational disease is enormous, resulting in over 60,000 deaths annually (Leigh et al. 1997) national estimates of the proportion specifically attributable to chemical exposures have not yet been compiled (Herbert and Landrigan 2000). Occupational disease reporting is generally suspected to underestimate true disease rates, given both the underdiagnosis of, and difficulty of accounting for, noninjury work-related illnesses (Herbert and Landrigan 2000). Immigrants, minorities, and lower-income communities typically bear a disproportionate burden of occupational chemical exposures and associated diseases (California EPA 2004; Pastor et al. 2002; Robinson 1991).

Occupational health data are more readily available in Europe. During the development of REACH, European Union research on potential occupational health benefits afforded by the regulation projected that the improved safety of workplace chemicals could prevent up to 40,000 cases of asthma annually (50% of occupationally related cases), an equal number of dermatitis cases, and 10,000 cases of COPD each year (Pickvance et al. 2005). The European Commission further estimated that REACH would prevent about 4,300 occupational cancers per year. In all, the commission estimated that the REACH regulation would save €50 billion ($60 billion) over a 30-year period in total occupational disease prevention (European Commission 2003).

#### Hazardous waste

The management and cleanup of hazardous waste is another externalized cost largely attributable to existing chemical technology choices. Each year, the United States spends more than $1 billion managing Superfund sites, and future costs are estimated at $250 billion (U.S. EPA 2007; U.S. GAO 2005b). On the current trajectory, the U.S. EPA anticipates the need for 217,000 new hazardous waste sites over the next 20 years (U.S. EPA 2004, 2007). This “end-of-pipe” approach to hazardous waste management can fail. In California, 70% of legacy sites are leaking directly into groundwater, having breached their containment, and are now posing what the state’s Department of Toxic Substances Control (2007) calls a major threat to human health or the environment. That half of the most prevalent chemicals at existing sites are known teratogens, neurotoxicants, and/or carcinogens raises serious concerns for the health of residents in communities surrounding these sites (Agency for Toxic Substances and Disease Registry 2003; Ostrowski et al. 1999).

### Toward a new chemicals policy

The cases noted above illustrate the expanding health and environmental problems attributable to an antiquated U.S. chemicals policy that has failed to keep pace with developments in either green chemistry or the environmental health sciences. Given the size of the chemical enterprise, the extent to which it is woven into the fabric of society, and the backlog of unexamined chemicals, a new approach is needed that does not rely on resource-intensive, chemical-by-chemical risk assessments in which government, at great public expense, bears the burden of proof. An integrated chemicals policy is needed that enables identification and prioritization of chemicals of concern and employs market and regulatory tools sufficient to motivate investment by industry in the design and production of safer chemicals and materials, based on the principles of green chemistry. Making this transition will require a chemicals policy that departs markedly from the federal policies of the last 30 years, of which TSCA is emblematic.

#### The link to green chemistry

The principles of green chemistry offer an upstream solution to many of the health, environmental, and economic problems related to industrial chemicals (Anastas and Warner 1998; DeVito and Garret 1998; Lancaster 2002; Lempert et al. 2003; NRC 2005). Implementation of these principles is critical if the chemical enterprise is to achieve sustainability. Specifically, green chemistry products are designed to be inherently less toxic and more readily broken down in the environment. Green chemistry processes use safer materials, operate more efficiently, and produce much less hazardous waste.

To date, producers have not invested in green chemistry at a level commensurate with the scale and pace of chemical production (Cone 2008a, 2008b; Iles 2006; LaMonica 2007; Lempert et al. 2003; NRC 2005). This is, to a large extent, a consequence of the U.S. chemicals market, which has been shaped over the last 30 years by the combined effects of the data, safety, and technology gaps. The European Union, meanwhile, has built a case for green chemistry in the European market (Black 2008; Henzelmann et al. 2007), raising the twin specters of the U.S. chemicals industry lagging behind its European counterpart in green chemistry innovation, and the United States becoming a market for hazardous chemicals and products that are prohibited for sale in the European Union and elsewhere (Cone 2006; Energy & Enviro Finland 2008).

The laws governing the chemical enterprise help define the incentives and disincentives that guide economic behavior in the market (Guth 2008). We use the term green chemistry in this context: as an analytical framework that encompasses both the science of safer chemistry and the laws and policies that will motivate its development and adoption by society. Although there are a variety of financial, technical, organizational, and cultural barriers to the widespread adoption of green chemistry practices by industry (Matus et al. 2007), addressing these barriers alone will not be sufficient to transform the chemical enterprise. Removing these barriers will require policy and regulatory reforms to improve the structure of incentives in the chemicals market.

## Mapping the Policy Gaps

Of the federal environmental statutes, TSCA is the only U.S. law intended to enable regulation of industrial chemicals both before and after they enter commerce. Other federal laws that pertain to chemicals are essentially “end-of-pipe” statutes that aim to control chemical emissions and exposures but do not permit premarket review of chemicals. Whereas TSCA potentially applies to some 83,000 chemical compounds, five other major U.S. statutes combined currently apply to only 1,134 chemicals and pollutants (Dernbach 1997) (Table 1).

TSCA was the U.S. response to conditions in which, before 1976, tens of thousands of chemicals entered markets without any form of accountability or oversight (Goldman 2002). Congress articulated the statute’s objectives in TSCA §2 (TSCA 1976):

- Chemical producers should develop adequate data on the health and environmental effects of chemical substances and mixtures.
- Government should have adequate authority to regulate chemical substances that present an unreasonable risk to health or the environment, and to take action on imminent hazards.
- The government’s authority over chemical substances should not create unnecessary economic barriers to technological innovation.

Based on these goals, TSCA promised to be an important step forward in the regulation of industrial chemicals. In practice, however, its legal and procedural requirements have largely thwarted these objectives.

## The Data Gap

### Logical paralysis

When it was passed in 1976, TSCA grandfathered the 62,000 chemical substances that were in commercial circulation at that time; that is, except on a case-by-case basis, chemical producers were not required to generate and disclose any information about the uses or hazard traits of these products (U.S. GAO 1994). In essence, this body of existing chemicals was assumed to be safe unless the EPA could prove otherwise. To gather the necessary hazard and exposure data from producers, however, TSCA §4 required the U.S. EPA to establish, on a chemical-by-chemical basis, *a*) that a substance may present an unreasonable risk to human health or the environment, or *b*) that there is either significant human exposure potential or substantial quantities of the chemical are produced, imported, and released into the environment (TSCA 1976). This created a logical paralysis for the U.S. EPA: To systematically assess risks of existing chemicals, the EPA needed hazard and exposure data that producers were under no obligation to provide, unless the EPA could first show that such an unreasonable risk might in fact exist or that high exposures were already occurring. This has proved all but paralyzing for the U.S. EPA. In the first 15 years under TSCA, the agency was able to review the risks of about 1,200 (2%) of the 62,000 existing chemicals, despite the fact that the agency estimated that about 16,000 (26%) were potentially of concern based on their production volume and chemical properties (U.S. GAO 1994). TSCA §8(e) does require that chemical manufacturers, processors, and distributors notify the U.S. EPA of any new or unpublished chemical hazard information, and the EPA receives about 300 such submissions each year (U.S. EPA 2003). Perversely, however, this requirement creates a disincentive for manufacturers to voluntarily investigate the hazard properties of their products.

### Existing chemicals

Although the TSCA inventory has grown to about 83,000 substances, the body of 62,000 existing chemicals still constitutes nearly all chemicals in commercial use in the United States. In the 2002 reporting year, 3,000 high production volume (HPV) chemicals—those produced or imported at more than one million pounds per year—made up > 99% by volume of the 15 trillion pounds of chemicals in commerce. Although these HPV chemicals constitute only one-third of existing chemicals by count, their high volume raises concern about the lack of basic hazard information on existing chemicals. Although the U.S. EPA’s New Chemical Program requires some minimal data on chemicals introduced since 1976, these chemicals make up < 1% of the production volume of the substances under the jurisdiction of TSCA (U.S. EPA 2005).

In tacit acknowledgement of its constraints, the EPA has turned to voluntary initiatives to close the data gap on existing chemicals. The HPV Challenge (U.S. EPA 2008b) is one such effort, begun in 1997 as an effort to gather screening-level data on HPV chemicals. It has been substantially limited, however, by late, incomplete, and poor- quality data submissions by chemical producers (Denison 2007; Denison and Florini 2004). In 2008, the U.S. EPA announced the Chemical Assessment and Management Program, in which the agency plans to conduct risk-based prioritizations for about 6,750 chemicals (U.S. EPA 2008a). These assessments, however, rely on incomplete data and information from TSCA inventory updates, much of which is obscured by trade secret claims and a lack of transparent assessment methods (Denison 2008).

## The Safety Gap

### Barriers to action

Given that the U.S. EPA bears the burden of proof, the agency is further constrained by the level of evidence required to take regulatory action. To regulate a chemical, TSCA §6 requires the U.S. EPA to provide substantial evidence of all of the following conditions: The chemical presents or will present an unreasonable risk to health and the environment; the benefits of regulation outweigh both the costs to industry of the regulation and the lost economic and social value of the product; the U.S. EPA has chosen the least burdensome means of addressing the source of unreasonable risk; and that no other statute could adequately address the risk (TSCA 1976).

Under this evidentiary burden, the U.S. EPA has been able, since 1976, to use its formal rule-making authority to partially regulate five existing chemicals (or chemical classes): polychlorinated biphenyls (PCBs), chlorofluoro carbons, dioxins, asbestos, and hexavalent chromium (U.S. GAO 1994). Of these, an amendment by Congress to TSCA required regulation of PCBs, and the U.S. EPA’s asbestos regulation, promulgated after the agency spent 10 years building its case, was overturned in its most significant aspects by the 5th Circuit Court of Appeals, which concluded that the U.S. EPA had failed to meet its burdens of proof under TSCA (Goldman 2002).

### New chemicals

TSCA does grant the U.S. EPA relatively more authority to regulate new chemicals introduced since 1976, as well as new uses of existing chemicals. Producers must submit premanufacturing notices (PMNs) before marketing a new chemical. However, there is no required minimum data set beyond information already in the possession of the producer at the time they file the PMN. This has created a disincentive for manufacturers to investigate their products’ potential hazards because, although known hazards must be reported, ignorance of hazard is not penalized. Not surprisingly, an EPA evaluation found that 85% of PMNs lacked data on chemicals’ health effects, and 67% lacked health or environmental data of any kind (U.S. EPA 2003). Hindered by limited data and the short time period permitted for the agency’s review, the U.S. EPA has taken some form of action on < 10% of the 36,600 chemicals that producers have proposed for commercial use between 1979 and 2004 (U.S. EPA 2007). Once new chemicals have entered commercial use, the U.S. EPA may regulate them only under the standards and burdens it carries for existing chemicals, as described above.

### Trade secrets

Extensive trade secret claims permitted under TSCA have exacerbated both the data and safety gaps. Although some protection of proprietary information is necessary, in practice the statute’s allowances for confidential business information (CBI) claims have severely limited access to basic information on chemical identity and use. In 2005, the U.S. EPA reported that 95% of PMNs submitted by producers contained some information claimed as confidential (U.S. GAO 2005a). One assessment found that 90% of the CBI claims in PMNs hid the identity of the chemical (U.S. EPA 2003). CBI allowances under TSCA have thus contributed to a pervasive lack of supply-chain transparency about chemical hazards, despite other regulations intended to facilitate hazard communication via Material Safety Data Sheets (Sattler et al. 1997; Occupational Safety and Health Administration 2004; U.S. Government Accountability Office 1991).

## The Technology Gap

### Minimal investment

By requiring less hazard information for existing substances than for new chemicals (data gap) and by stymieing effective regulation of well-known hazards (safety gap), TSCA has produced a chemicals market that favors existing chemicals over newer and potentially less toxic substances. As a result, the statute provides little motivation for industry investment in green chemistry research and development. The vast majority of chemical products manufactured in the United States rely on technologies developed 40–50 years ago, a fact that led the Council for Chemical Research to call for new technologies that incorporate economical and environmentally safer processes, use less energy, and produce fewer harmful byproducts (Council for Chemical Research 1996). Twelve years after the Council’s *Vision 2020* report, the Web sites of the 50 largest U.S. chemical companies all state their commitment to reaching sustainability goals, but their spending on research and development has remained level or declined since about 2000 (Lempert et al. 2003; NRC 2005). A National Research Council report concluded that this trend makes it difficult to advance the science and technology needed to support such sustainability goals (NRC 2005).

Public investment is also critical to the development of green chemistry. The U.S. experience in other sectors illustrates that investment by government, federal laboratories, and research universities helps spur innovation of new technologies (Block and Keller 2008).

### Education and the market

It is a reflection of the priorities of the U.S. chemicals market that, with rare exception, students can earn an undergraduate or graduate degree in chemistry at universities throughout the United States without demonstrating an understanding of the principles of toxicology, ecotoxicology, or green chemistry (Cone 2008c; NRC 2007). Educational priorities have largely matched those of the chemicals market, such that the hazard traits of a substance, for example, are undervalued in the chemistry classroom relative to chemical function, price, and performance. Without a policy strategy that will favor green chemistry in the market, and without a corresponding research and educational effort, the United States risks lagging behind the European Union and other industrialized regions in the scientific and technical development of green chemistry.

## Effects of the Three Gaps

The most striking implication of the data gap is that the U.S. EPA lacks the information it needs to identify potential threats to public health and the environment. Perhaps equally significantly, the data-poor market makes safer alternatives difficult to distinguish from hazardous chemicals, distorting market signals (Guth et al. 2007). Furthermore, chemical hazard information generated by producers is often asymmetrically distributed, with inadequate communication to the market. Given better information, many downstream businesses, as well as governments, consumers, and workers, would be better able to express a preference for safer chemicals and products. The ability to identify safer substances could potentially lower the business costs of handling hazardous substances, estimated at 7–10 times the purchase cost of the chemical (Chemical Strategies Partnershp 2004).

The safety gap compounds these problems by allowing the commercial circulation of hazardous substances. The high level of evidence TSCA requires of the U.S. EPA makes it difficult for the agency to act on information it does have and impose restrictions on chemical use. With the burden of proof on government, lack of information increases the likelihood that hazardous chemicals will not be regulated. Some have argued that what begins as a regulatory disincentive for producers to generate or disclose hazard information has become an incentive to create misleading information that casts doubt on scientific evidence (Michaels 2008). Given the high evidentiary threshold, doubt, in and of itself, effectively impedes the agency’s ability to take action (Guth et al 2007).

Finally, as a result of the incentives created by TSCA, chemical producers are rationally motivated to defend existing chemicals (Ashford 2000; Echikson 2006). Those invested in existing chemicals have a strong commercial interest in resisting policies that could improve market transparency and the commercial viability of safer substitutes, as evidenced by the efforts of the American Chemistry Council to influence REACH negotiations (Brown 2003; Loewenberg 2006; U.S. House of Representatives 2004).

Although large “sunk” investments in existing chemicals and processes could make it difficult to transform the industrial system to one based on the principles of green chemistry, industry will have to make this transition if the United States is to meet the challenge of economic and environmental sustainability.

## Closing the Gaps: Implications for the Environmental Health Sciences

The transition to a sustainable chemical enterprise in the United States will require a fundamental reform of TSCA that meets three overarching objectives:

- Close the data gap: Provide for the effective operation of the chemicals market by requiring that chemical producers generate, disclose, distribute, and effectively communicate sufficient information to stakeholders on the hazard properties of chemicals.
- Close the safety gap: Provide government with the legal tools necessary to identify, prioritize, and take action to reduce chemical hazards and exposures.
- Close the technology gap: Build capacity in cleaner chemicals and processes by incorporating scientific, technical, legal, and policy-related elements of green chemistry into the nation’s education and research infrastructure.

Accomplishing these objectives will require both supply-side and demand-side strategies (Green Chemistry Initiative 2008). Supply-side strategies address the technology gap. They are intended to improve the supply of the science, technology, and commercial applications of green chemistry through advancements in education, research, and development. Demand-side strategies address the data and safety gaps, primarily through public policies to drive data generation and disclosure and to regulate known hazards, a combination that ultimately improves the structure of incentives in the chemicals market. Demand-side policies also include, for example, laws that extend the scope of producer responsibility to include the complete product life cycle.

The two approaches operate in tandem: Demand-side strategies provide the requisite drive for supply-side solutions by generating the market need for new science and technology. That is, demand stimulates the private and public investments necessary to advance green chemistry innovation. The importance of market demand as a driver for industrial innovation is well established in the environmental sector. A survey of the executives of 90 leading companies operating in 13 European Union countries identified public policy and market demand as the two most important factors necessary for motivating environmental innovation in their companies (Henzelmann et al. 2007). Recent reports by the U.S. GAO (2009) and the Environmental Defense Fund (Denison 2009) are among others that have called for policy changes to improve the incentive structure in the chemicals market by, for example, strengthening the U.S. EPA’s authority to obtain chemical hazard information from producers and shifting more of the burden to producers to demonstrate the safety of their products.

Undertaking meaningful chemicals policy reform in the United States will engender new research questions that must be informed by the environmental health sciences. Through the lens of the three policy gaps, these questions include the following:

### The data gap

- What is the most useful and attainable body of standardized hazard and exposure information that should be generated for chemicals, and how should a chemical’s sales volume and inherent hazard traits drive the scope of data requirements?
- What is the proper role of producers in generating these data, and how can the credibility, standardization, and quality of these data be assured?
- In what ways can emerging predictive toxicity testing and exposure methods be applied to meet these data needs?
- What information on chemical hazards, exposures, and uses if made publicly available would most effectively protect public and environmental health and motivate the development of safer alternatives?
- What are the most effective means of communicating chemical hazard and exposure information to stakeholders, including product formulators, downstream businesses, communities, workers, consumers, and government agencies?

### The safety gap

- On what measures of hazard and exposure (such as environmental persistence, bioaccumulative potential, toxicity, or presence in consumer products) should chemicals be prioritized and safer alternatives defined?
- To what extent (and in what ways) should producers carry the burden of proof of chemical safety?
- What level of evidence of potential harm to health or the environment is sufficient to trigger government action?
- What portfolio of actions should government employ to efficiently address identified hazards?
- What are the appropriate bounds of producer responsibility over the life cycle of chemicals and products, and what policies can best ensure producer responsibility within these bounds?

### The technology gap

- How can developments in the environmental health sciences (such as biomonitoring findings or the science of endocrine-disrupting chemicals) best be communicated to the chemical enterprise so as to drive continuous improvement in chemical design?
- What are the high-priority chemicals and processes that warrant publicly funded research into green chemistry alternatives?
- What are the scientific, technical, and practical barriers to implementing these alternatives, and how are these barriers best addressed?
- How should green chemistry inform the development of next-generation environmental technologies, such as alternative energy and building materials, to reduce their health and environmental impacts and improve their overall sustainability?
- How should green chemistry education be designed to better prepare scientists, engineers, and decision makers to respond to the challenges of sustainability?

## Conclusion

Although some leading businesses have adopted green chemistry methods, the vast potential of green chemistry remains untapped. This primarily reflects the priorities of the U.S. chemicals market, in which chemical safety is undervalued relative to function, price, and performance. Hazardous chemicals have thus remained competitive, despite the many costs society bears as a result of their production, use, and eventual disposal. These market conditions are a consequence of the chemical data gap, safety gap, and technology gap that have grown out of weaknesses in the language and implementation of TSCA. A new U.S. chemicals policy has the potential to address the societal costs—human, environmental, and economic—that have accompanied advancements in the chemical enterprise.

New chemical and product laws in the European Union have opened an opportunity for chemicals policy reform in the United States. A fundamental restructuring of TSCA will need to simultaneously correct the data, safety, and technology gaps using strategies that improve both the demand for and supply of green chemistry technologies. The attendant research questions demand engagement from the environmental health sciences, and their solutions offer the possibility of improving human health and environmental protection while moving the United States to a position of global leadership in green chemistry innovation.

## Figures and Tables

**Figure 1:**
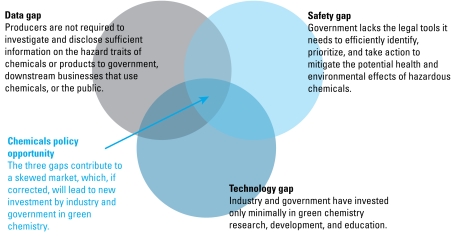
The three gaps in U.S. chemicals policy. Policy measures that address the gaps will promote sustainable innovation in the chemical enterprise while improving human health and the environment.

**Table 1:** Numbers of industrial chemicals and pollutants governed by U.S. law, excluding TSCA.

Federal statute	No. of substances
Clean Water Act (1966)	148
Resource Conservation and Recovery Act (1976)	502
Clean Air Act (1963)	189
Occupational Safety and Health Act (1970)	453
Emergency Planning and Community Right-to-Know Act; Toxics Release Inventory (1986)	600

Although TSCA applies to tens of thousands of substances, only 1,134 chemicals and pollutants are listed under five major federal statutes (with overlap) (Dernbach 1997).
